# Transient dynamics between displaced fixed points: an alternate nonlinear dynamical framework for olfaction

**DOI:** 10.1186/1471-2202-12-S1-P237

**Published:** 2011-07-18

**Authors:** Christopher L Buckley, Thomas Nowotny

**Affiliations:** 1 CCNR, Informatics, University of Sussex, Falmer, BN1 9QJ, UK

## 

It is now widely accepted that insects encode odor identity in terms of the stimulus specific rate patterning observed in the projection neurons of the antennal lobe (AL). Several studies have explored nonlinear dynamical frameworks in order to gain general insights into the mechanisms underlying the observed dynamical phenomena. The currently most popular framework is the heteroclinic orbit (HO) developed by Rabinovich and collaborators [1-3]. This framework can account for both the dynamics observed in the locust AL [4] and several desirable computational properties of the AL and the brain in general [2].

Despite its obvious appeal there has been little work on applying the HO framework to more detailed conductance based models of olfactory dynamics even though it is in principle possible to obtain HO dynamics from biologically plausible neurons [5]. However, obtaining proper HO dynamics with conductance based Hodgkin-Huxley neurons in this work required non-standard assumptions for the synapses [5]. Furthermore, recent experimental evidence has indicated another possible concern about the original HO interpretation. The HO framework assumes that the presence of an odor re-parameterises the connectivity of the AL through input to local neurons (LNs). The mathematical image of this change is a bifurcation from a regime with a single stable fixed point (FP) to a regime with a stable HO. The system approaches the stable HO (or a limit cycle close to it, in the presence of noise) as long as the odor stimulation continues. The HO then loses stability and the system returns to the resting state FP when the odor stimulus subsides. Recent experiments involving long periods of odor stimulation have revealed that the dynamics of the AL appears to settle into a stable fixed point well before the odor subsides [6]. One explanation for this observation is the so called heteroclinic channel (HC) introduced in [2] in which the HO ends in a stable FP.

Here we describe an alternate nonlinear dynamical framework which provides an appealing account of both the origin and function of rate patterning dynamics. We start by analytically reducing a biologically plausible conductance based model of the AL to a quantitatively equivalent rate model and construct conditions such that the rate dynamics are well described by a single globally stable fixed point. We then straightforwardly describe the AL's response to an odor stimulus as rich transient trajectories between this stable baseline state and an odor specific fixed point. We show how this framework can account for a disinhibitory period observed immediately after the stimulus is removed, for the qualitative differences between the dynamics in the presence and the absence of odor and for the invariance of odor identity to both the duration and intensity of an odor stimulus, as reported in [7]. We compare and contrast this framework with the popular nonlinear dynamical framework of 'winnerless competition' which describes AL dynamics in terms of a HO.
